# Overcoming immunotherapy resistance in non-small cell lung cancer (NSCLC) - novel approaches and future outlook

**DOI:** 10.1186/s12943-020-01260-z

**Published:** 2020-09-11

**Authors:** Lena Horvath, Bernard Thienpont, Liyun Zhao, Dominik Wolf, Andreas Pircher

**Affiliations:** 1 Internal Medicine V, Department of Hematology and Oncology, Medical University Innsbruck, Anichstraße 35, 6020 Innsbruck, Austria; 2 Laboratory for Functional Epigenetics, Department of Human Genetics, KU Leuven, Herestraat 49, 3000 Leuven, Belgium; 3 Medical Clinic III, Department of Oncology, Hematology, Immunoncology and Rheumatology, University Hospital Bonn (UKB), Sigmund-Freud-Street 25, 53127 Bonn, Germany

**Keywords:** NSCLC, Immunotherapy resistance, Tumor microenvironment heterogeneity, Targeted therapy

## Abstract

Immunotherapy (IO) has revolutionized the therapy landscape of non-small cell lung cancer (NSCLC), significantly prolonging the overall survival (OS) of advanced stage patients. Over the recent years IO therapy has been broadly integrated into the first-line setting of non-oncogene driven NSCLC, either in combination with chemotherapy, or in selected patients with PD-L1^high^ expression as monotherapy. Still, a significant proportion of patients suffer from disease progression. A better understanding of resistance mechanisms depicts a central goal to avoid or overcome IO resistance and to improve patient outcome.

We here review major cellular and molecular pathways within the tumor microenvironment (TME) that may impact the evolution of IO resistance. We summarize upcoming treatment options after IO resistance including novel IO targets (e.g. RIG-I, STING) as well as interesting combinational approaches such as IO combined with anti-angiogenic agents or metabolic targets (e.g. IDO-1, adenosine signaling, arginase). By discussing the fundamental mode of action of IO within the TME, we aim to understand and manage IO resistance and to seed new ideas for effective therapeutic IO concepts.

## Background

Immunotherapy (IO) and particularly immune checkpoint inhibitors (ICI), including programmed death receptor 1 (PD-1) and PD-ligand 1 (PD-L1) inhibitors have revolutionized the treatment landscape of non-small cell lung cancer (NSCLC). Previously unanticipated long-term responses in advanced stage disease have been accomplished, with a 5 year overall survival (OS) of 20% in unselected and up to 40% in PD-L1^high^ expressing patients [1].

Despite the striking clinical improvements, the majority of patients eventually fails to respond to ICI therapy due to the evolution of primary or secondary resistance. Prospective clinical studies to demonstrate treatment strategies following progression on IO therapy are still lacking.

Various IO resistance mechanisms have been characterized, involving tumor cell intrinsic as well as environmental resistance patterns. The tumor microenvironment (TME) plays a critical role by influencing both extrinsic and intrinsic resistance pathways. A better understanding of the heterogenous TME will set stage for further optimizing strategies and guide new avenues in future IO treatment stratification.

This review discusses the multitude of novel preclinical and clinical treatment approaches that aim to overcome IO resistance in NSCLC. The complexity of cellular and molecular alterations within the immunosuppressive TME build the fundament for designing rational and synergistic combination therapies that lower the risk of resistance and prolong benefit from IO therapy.

## Immunopathology of NSCLC and evolution of IO resistance

**Fig. 1:**
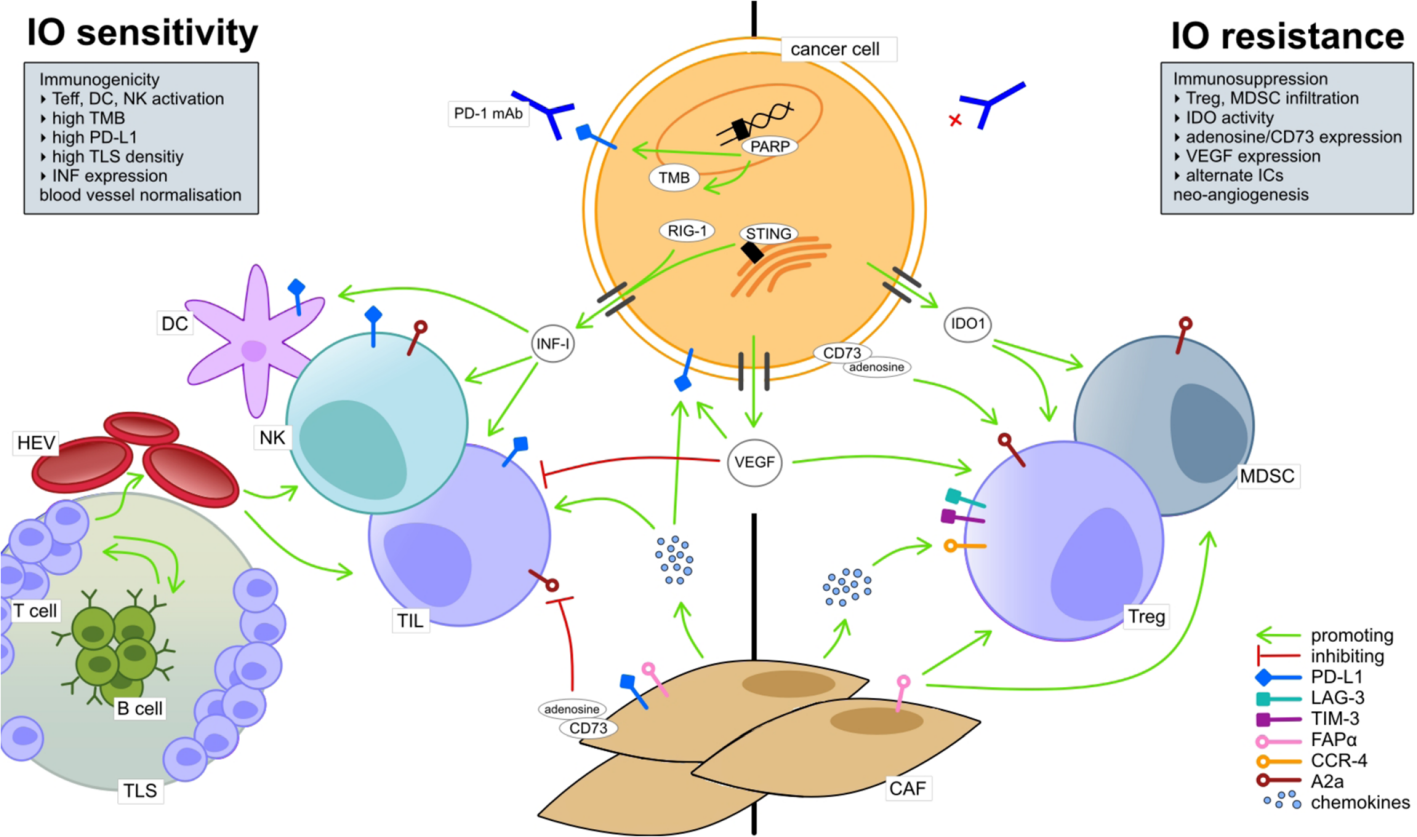
Overview of the cellular TME composition and major molecular pathways associated with IO sensitivity (left) and resistance (right). IO sensitivity is depicted by an immunogenic TME, comprising the activation of effector immune cells (e.g. tumor infiltrating lymphocytes (TIL), dendritic cells (CD) and natural killer cells (NK)). Naïve T cells undergo activation and priming in close association to B cells within tertiary lymphoid structures (TLS). T effector cells transmigrate to the stromal TME compartment via high endothelial venules (HEV), tightly regulated by immunomodulatory tumor endothelial cells (TEC; not illustrated) in the HEV endothelium. Cancer cell intrinsic molecular pathways that enhance TME immunogenicity involve interferon type I (IFN I) expression, which is, amongst other stressors, induced by cytosolic RIG-I or by an activated STING pathway. IO sensitivity is enhanced in a TME with high PD-L1 expression by cancer and immune cells. High neo-antigen expression by cancer cells as result of high tumor mutational burden (TMB), e.g. induced by PARP inhibition, enhances TME immunogenicity and IO sensitivity. IO resistance is marked by an immunosuppressive TME and includes, on a cellular basis, infiltration of T regulatory cells (Treg) and myeloid derived suppressor cells (MDSC) as well as M2 macrophages (not shown). CD73 and, thus, adenosine expression by cancer cells or fibroblasts leads to inhibition of TIL and promotion of Treg; CD73 upregulation associates with cancer immune evasion. Also, up-regulation of alternative immune checkpoints e.g. LAG-3 and TIM-1 by immune cells enhances IO resistance. Cancer associated fibroblasts (CAF) depict both immunosuppressive and immunostimulatory functions, e.g. via chemokine release. Upregulation of the chemokine receptor CCR-4 is associated with IO resistance. Vascular endothelial growth factor (VEGF) gets ubiquitously expressed in the TME (not illustrated, see Fig. 2). It has immunosuppressive functions by inhibiting effector immune cells (e.g. TIL, NK, DC), upregulating inhibitory immune checkpoints (e.g. PD-L1) and by promoting Treg and MDSC. Tumor growth promoting neo-angiogenesis (not illustrated) is driven by hypoxia and, thus, VEGF expression

IO resistance mechanisms result from the constantly evolving interactions between cancer cells and the surrounding cell populations within the TME, including immune cells, cancer-associated fibroblasts (CAF) and tumor endothelial cells (TEC) (Fig. 1). The following section recapitulates the basic characteristics of the immunogenic TME, particularly focusing on IO response- or resistance-mediating mechanisms and biomarkers.

### Immune checkpoints

**Fig. 2:**
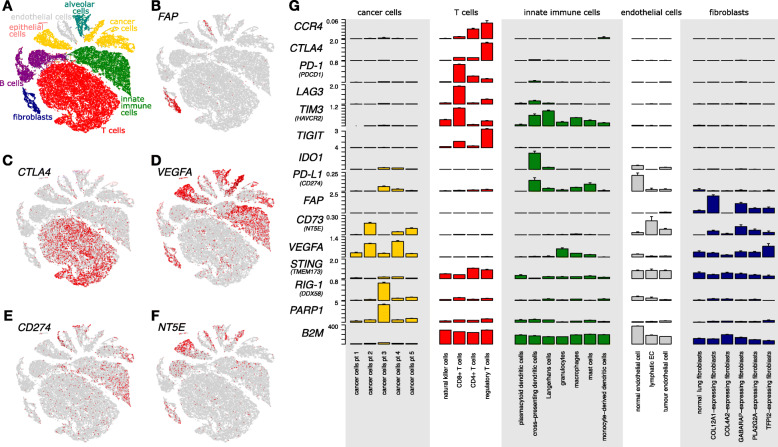
The gene expression heterogeneity of the NSCLC TME, illustrated by gene expression in stromal and cancer cells. 52.698 single cells from 4 non-malignant and 15 tumor samples of five patients were analyzed. **(a-f)** tSNE plots of the 52.698 cells, with (**a**) clusters color-coded according to the associated class of cell types, or with (**b-f**) cells colored according to the expression of the indicated marker gene, illustrating the heterogeneity of gene expression by the various cell types within the TME. Gene expression is shown ranging from grey to red (low to high). **(e)** CD274 is the gene alias for PD-L1. **(f)** NTE5 is the gene alias for CD73. **(g)** Expression levels of selected genes (gene alias in brackets), involved in immunomodulation in tumors shown separately for each cell type based on single cell RNA sequencing. Expression levels in cancer cells are shown separately for each patient, while the subtypes of T cells, innate immune cells, endothelial cells and fibroblasts represent pooled patient data. Data are derived from reference [2]

Immune checkpoints (IC) play a central role in negative regulation of T cell reactivity and their inhibition via monoclonal antibodies can unleash T cell-triggered antitumor immune responses. The best studied IC are PD-1 and cytotoxic T lymphocyte antigen 4 (CTLA-4). PD-1 is broadly expressed on CD8^+^ T lymphocytes, regulatory T cells (Treg) and natural killer (NK) cells and modulates T cell activity via interaction with its ligand (PD-L1) in the TME (Fig. 2). CTLA-4 is expressed on CD8^+^ and CD4^+^ T lymphocytes and Treg and regulates early naïve T cell activation in secondary lymphoid organs [3, 4]. Other IC are constantly being discovered and under investigation for their clinical utility as druggable IC (e.g. TIM-3, LAG-3 or TIGIT).

### Lymphocytes of the tumor microenvironment

#### T lymphocytes

Tumor infiltrating T lymphocytes (TIL) play a major role in antitumor immune responses within the TME [5]. The phenotype of T cell infiltration varies strongly between different tumors in terms of quantity and distribution and is associated with IO efficacy. Classifying tumors based on the cytotoxic T cell infiltration phenotype might help to rationally guide treatment stratification [5].

#### Tertiary lymphoid structures

In chronically inflamed areas such as tumors, B and T lymphocytes are frequently organized in ectopic lymphoid aggregates, so-called tertiary lymphoid structures (TLS), where they convert to effector cells upon antigen presentation. The cellular organization ranges from simple lymphocyte clusters (immature TLS) to highly sophisticated structures (mature TLS) [6, 7]. High endothelial venules (HEVs) are found nearby and promote lymphocyte extravasation [8]. TLS display a surrogate marker of prompt immune responses that actively modulate anticancer immunity [7]. High TLS density associates with a favorable prognosis in many cancer types, including NSCLC [9] and TLS may also enhance IO efficacy [6]. Preclinical studies demonstrated beneficial effects of therapeutic TLS neogenesis on anti-cancer immune responses [10–12].

#### B lymphocytes

Tumor infiltrating B cells harbor both immunostimulatory [13] and immunosuppressive [14] functions and their effect on IO efficacy is increasingly appreciated. Especially those B cells located in mature TLS may exhibit immunostimulatory functions by closely interacting with local T cells, thereby enhancing anti-cancer immunity. This hypothesis is indirectly supported by the observation that intra-tumoral B cells are linked to a favorable IO response [7, 15, 16].

### Tumor Mutational Burden (TMB)

Somatic mutations in the cancer genome, such as in DNA repair genes including mismatch repair (MMR), homologous recombination (HR) or polymerase epsilon (POLE) increase tumor mutational and neoantigen burden, which has been linked to greater TIL density and enhanced ICI efficacy [17–19]. This observation is clinically underscored as mutagen-driven cancer types (e.g. melanoma, NSCLC) typically show high initial ICI responses [17]. Moreover, components of the major histocompatibility complex I (MHC I) such as B2M are often downregulated (Fig. 2), hence curbing neo-epitope presentation to T cells [20]. Antigen presentation pathways can also be inactivated through mutations (e.g. B2M is mutated or deleted in about 5% of lung cancers) [21] and also other pathway members are inactivated [22]. Importantly, IO may increase the frequency of such mutations [19, 23, 24] suggesting an active immune-editing of cells failing to present neo-epitopes.

Concerning TMB as predictive biomarker of ICI response, clinical trials report divergent results, possibly due to technical issues with TMB assessment (e.g. use of inhomogenous cut-off values) [25]. On the one hand, high TMB was the strongest variable linked to benefit of combined PD-1 plus CTLA-4 blockade in NSCLC and TMB was independent of PD-L1 expression [26]. Accordingly, pembrolizumab was recently FDA-approved in TMB^high^ advanced solid cancers (≥10 mutations/megabase) in response to results from KEYNOTE-158. In contrast, in the complex multi-arm CheckMate227 trial testing ipilimumab *plus* nivolumab versus chemotherapy or nivolumab *plus* chemotherapy in NSCLC, neither TMB nor PD-L1 expression could segregate therapy responsiveness [27]. Concerning CTLA4-specific biomarkers, different genomic signatures were correlated with enhanced clinical outcome [28, 29], however none have been translated into clinical practice yet.

### PD-L1 expression in the TME

Cancer cells can overexpress PD-L1 upon type I interferon (IFN I) stimulation [30] to evade cytotoxic immune responses. Immune cells, including Treg, myeloid-derived suppressor cells (MDSC), dendritic cells (DC) and TEC can similarly upregulate PD-L1 upon inflammatory signals (especially by IFNs) fostering an immunosuppressive TME [31]. Interestingly, myeloid cells show markedly higher PD-L1 expression than cancer cells or lymphocytes (Fig. 2) and especially extra-tumoral PD-L1 expressing myeloid cells, e.g. in tumor draining lymph nodes, might be essential for ICI response [31]. A preclinical study demonstrated that myeloid progenitors that accumulate during cancer-driven emergency myelopoiesis (in bone marrow, spleen and tumor site) show both PD-L1 and particularly prominent PD-1 expression. Selective deletion of myeloid-specific PD-1 by targeting the *Pdcd1 gene* effectively suppressed tumor growth in several tumor models by mediating antitumor immunity (enhanced T effector memory cells) despite preserved T cell-specific PD-1 expression. These data underline the important role of myeloid-intrinsic effects in regulating anti-tumor immunity [32].

Clearly, PD-L1 expression is necessary to achieve adequate responses to PD-1/PD-L1 blockade and numerous studies associated high tumor cell PD-L1 expression with better outcomes to anti-PD-1/PD-L1 monotherapy in NSCLC. Controversially, some patients with very low or even absent PD-L1 expression show durable responses [33], an observation currently lacking a mechanistic explanation see 2.4.1. Besides cancer cells, also PD-L1 positive immune cells may exert a predictive value. In the Impower110 trial, presence of PD-L1 positive TIL significantly associated with enhanced OS in patients treated with atezolizumab [34]. These results are in line with other tumor entities (e.g. bladder and breast cancer).

#### PD-L1 is not yet a robust biomarker

So far, clinical trials considered tumor PD-L1 expression as the most robust and reproducible biomarker, and clinical NSCLC guidelines are based on this. However, PD-L1 immunohistochemistry (IHC) has several limitations (e.g. biopsies from primary versus metastatic lesions, different detection antibodies and cut-offs, staining procedures) and this may contribute to the above-mentioned controversial observations. Moreover, the TME is highly heterogenous and a single core biopsy only depicts one spatial tumor component, hence some patients may be PD-L1 negative in one biopsy and PD-L1 positive in other tumor areas. This also explains quantification errors in tissue-based biomarkers. One approach to resolve the limitation of spatial resolution involves PET-based PD-L1 imaging with zirconium-89-labeled atezolizumab. Interestingly, pre-treatment tumor PET signal was shown to better correlate with clinical treatment responses than IHC or RNA-sequencing based predictive biomarker-detection [35].

### Tumor-associated macrophages

Tumor-associated macrophages (TAM) are an abundant cell type within the TME and despite growing research, their role in cancer progression remains ambiguous. Along a functional scale, TAM polarize to either M1 or M2 phenotypes in response to environmental cues, including metabolic changes (e.g. cyclic hypoxia, nitric oxide) [36, 37]. The classically activated M1 phenotype is stimulated upon type 1 T helper cell (Th1)-produced IFN-γ or Toll-like receptor (TLR) ligands such as microbiota-derived lipopolysaccharide (LPS) and is characterized by phagocytic, cytotoxic and antigen-presenting functions and secretion of pro-inflammatory cytokines (e.g. TNFα, IL-1β, IL-6) [36, 38]. Alternatively, the M2 phenotype expands in response to Th2-derived IL-4 and IL-13 [39], but cancer cell-derived macrophage-colony stimulating factor (M-CSF) also promotes M2 polarization by binding CSF1 receptor (CSF1-R). M2 macrophages express anti-inflammatory cytokines (e.g. IL-10, CCL22, CCL18) and low levels of IL-12, thereby exerting anti-inflammatory, angiogenic and pro-tumoral effects [36]. Impeding M2 polarization to promote anti-tumor immune responses has gained clinical interest (e.g. CSF1 inhibition) and also preclinical studies of genetic TAM reprogramming are promising [40, 41].

### Cancer-associated fibroblasts

Cancer associated fibroblasts (CAF) constitute one of the most prominent, yet highly heterogenous components of the TME. They express a variety of molecular markers, e.g. α-SMA, S100A4, FAP, PDGFRα/β, none of which, however, is unique for the fibroblast lineage. Next to immune cells CAFs have emerged as important mediators of the complex stroma-tumor interactions, promoting local immunosuppression and orchestrating immune cell trafficking [42]. CAFs may express PD-L1 (e.g. upon IFN-γ) (Fig. 2) but may also promote PD-L1 expression on tumor cells via cytokine secretion (e.g. CXCL5, CXCL2) [43]. Further knowledge on CAF functionality might unveil insights in IO sensitivity.

### Tumor endothelial cells

Tumor endothelial cells (TEC) have immunomodulatory functions by controlling immune cell transmigration, lymphocyte activation and function. They hold a “sentinel” role in detecting foreign antigens as antigen (cross)-presenting cells, though this has been studied extensively in non-malignant inflammation and less in TEC [44, 45]. TEC are strategically positioned at the blood–TME interface, serving as “immune gatekeepers” by controlling immune cell trafficking. In NSCLC, TEC may express PD-L1 (Fig. 2) and downregulate inflammatory pathways (e.g. antigen presentation, chemotaxis, immune cell homing) [2]. On the single cell level, Goveia et al. identified distinct lung TEC subpopulations carrying the transcriptome signature of HEVs and semi-professional APCs, suggesting a role in tumor immune surveillance. Specific TEC subtypes were associated with prognosis and response to anti-angiogenic therapy [46].

### Resistance mechanisms

It remains to be answered why some patients attain sustained durable IO therapy response while others evolve primary or secondary resistance. The mode of action is definitely multifactorial and includes intrinsic (e.g. cell signaling, immune recognition, gene expression, DNA damage response) and extrinsic (e.g. T cell activation, neo-angiogenesis) mechanisms [47]. The following sections briefly address relevant resistance mechanisms, many of which are already used as targets of novel therapeutic strategies as to overcome resistance (see Fig. 1).

#### Intrinsic cancer cell resistance: immunogenicity

Neo-antigen burden of cancer cells markedly determines tumor immunogenicity, which enhances ICI efficacy. Hence, low tumor immunogenicity may cause primary IO resistance. Immune-cancer cell interactions can promote the evolution of low-immunogenic and low-antigenic tumor subclones, a process named immuno-editing [48]*.* Genetic instability due to impaired DNA repair can enhance tumor immunogenicity, which is the target of later discussed PARP inhibitors [47].

#### Intrinsic T cell resistance: Immuno-adaption

In response to PD-1/PD-L1/CTLA-4 inhibition, T cells can upregulate alternative ICs, including T cell immunoglobin mucin-3 (TIM-3) or lymphocyte activation gene 3 (LAG-3), as adaptive resistance mechanism [49, 50]. Co-expression of multiple ICs associates with severe T cell exhaustion, consequently leading to IO resistance [51].

#### Extrinsic resistance: Treg and MDSCs

An immunosuppressive TME facilitates tumor cell growth and tumor infiltrating Treg and MDSC are key players in sustaining this immunosuppression [52]. IO efficacy has been linked to lower Treg and MDSC infiltration in preclinical studies [53–55]. Moreover, Indoleamine 2,3-dioxygenase (IDO) represents an important promotor of Treg and MDSC proliferation/activation [56].

#### Extrinsic resistance: the chemokine milieu

Chemokines mediate immune cell recruitment into the TME and directly impact cancer and endothelial cells to regulate tumor cell proliferation, neo-angiogenesis and hence cancer progression. Multiple chemokines (four major subgroups CC, CXC, CX3C, C) have been identified with multi-faceted roles, acting both pro- or anti-cancerogenic in different tumor entities. Their impact on IO resistance and efficacy remains unclear [57].

#### Extrinsic resistance: VEGF

Vascular endothelial growth factor (VEGF) expression within the TME is heterogenous (Fig. 2) and mainly hypoxia-driven. VEGF is the key driver of tumor neo-angiogenesis but also exerts immunosuppressive effects [58]. Accordingly, anti-PD-1 non-responders showed higher VEGF levels compared to responders, suggesting a potential role of VEGF in IO resistance [59]. This at least partly explains potential additive and even synergistic effects of anti-VEGF and IO strategies, as described later.

## Future IO treatment strategies

The treatment landscape of non-oncogene driven NSCLC has changed dramatically in recent years and IO is an important cornerstone of front- and later-line therapies (we refer to the latest ESMO and ASCO guidelines [60, 61]). Yet, IO resistance occurs frequently, thus stressing the need for better therapy allocation based on predictive biomarkers. The cellular and molecular heterogeneity of the TME sets the stage for innovative prediction models in diagnostics and depicts a pivotal target of many tailored therapy approaches that aim to overcome IO resistance.

Multiple clinical trials in different cancer types are based on an exploding number of preclinical studies using novel IO combinations or targeted therapies. The following section will discuss the background, mode of action and clinical update of the most relevant up-coming treatment options in IO-refractory NSCLC.

### IO combination or re-challenge

IC co-inhibition, by expanding the anti-PD-1 or PD-L1 backbone with a second ICI has been one of the first strategies to overcome IO resistance and most clinical experience has been gathered with combinational CTLA-4 inhibitor. The observed synergistic effect of PD-1/CTLA-4 inhibitors likely depends on the distinct patterns of PD-1 and CTLA4 in immune activation, as PD1 blockade inhibits peripheral and CTLA4-blockade central tolerance see 2.1, 3.

#### Clinical experience of IO combination

The combination of CTLA-4 and PD-1 inhibitors is effective in melanoma [62] and renal cell carcinoma (RCC) [63] patients, having led to to FDA approval. In NSCLC, CheckMate227 demonstrated a prolonged OS benefit for first-line ipilimumab *plus* nivolumab in advanced-stage disease (median OS 17.1 vs. 13.9 months with chemotherapy, 2-year OS of 40% vs. 32.8% (HR 0.79, 97.72% CI 0.65–0.96; *P* = 0.007)), independent of TMB or PD-L1 expression. Intriguingly, the OS effect was most prominent in PD-L1^low^ patients. Treatment-related serious adverse events (AE) of any grade were more frequent with ipilimumab *plus* nivolumab than with chemotherapy (24.5% vs. 13.9%) [27].

Recent results from the phase II CITYSCAPE trial showed a significant PFS and ORR benefit for the first-line combination of the TIGIT-inhibitor see 3.1.4 tiragolumab *plus* atezolizumab compared to atezolizumab monotherapy in PD-L1 positive metastatic NSCLC patients. Particularly, a meaningful ORR improvement was seen in PD-L1^high^ (TPS > 50%) expressing patients (55.2% vs 17.2%) [64], while toxicity was not aggravated.

These data emphasize the potency of IO combination, but optimal patient selection criteria are still lacking.

#### IO re-challenge

In recent years, the dogma of disease progression being synonymous for drug resistance has been questioned [65], therefore re-challenging IO after progression displays a possible strategy.

Retrospective studies have investigated IO re-challenge in a small number of NSCLC patients with clinical benefit in only a minority of them [66–69]. Recently, a retrospective study including 10.452 NSCLC patients demonstrated the effectiveness of nivolumab retreatment after either treatment interruption or interim chemotherapy. OS in the retreatment situation significantly correlated with duration of initial IO exposure, which may be due to a time-dependent consolidation of an immune memory. The median OS for IO retreatment was above 12 months, which compares favorably with OS during initial nivolumab treatment or with standard third-line chemotherapy in advanced NSCLC [70]. Moreover, the phase III KEYNOTE-024 trial demonstrated the feasibility of a second course pembrolizumab in 10 NSCLC patients who had progressed after completion of 2 year pembrolizumab monotherapy, with an objective response rate (ORR) in 7/10 patients [71].

The question of dual ICI following IO progression has currently been investigated in two RCC studies. A small retrospective study (*n* = 17) could not show a substantial benefit of nivolumab *plus* ipilimumab after progression on first-line nivolumab [72]. Contrarily, the phase II TITAN trial (*n* = 207) showed a significant ORR benefit for the “immunotherapeutic boost” with 2–4 cycles of nivolumab *plus* ipilimumab in the first-line as compared to nivolumab monotherapy [73].

#### IO beyond progression

The discussion of continuing IO therapy beyond progression originates from the observation of initial pseudoprogression preceding objective response. However, pseudoprogression is rare (less than 10% of NSCLC patients) and hence IO continuation should only be considered in patients with clinical benefit and lack of severe AE [74]. Some NSCLC patients treated with ICI might present with dissociated response, where some tumor areas progress while others regress. Similarly to oligometastatic disease, a concomitant local treatment approach (radiotherapy, surgery) of the resistant clones could be discussed as possible option [75].

#### Alternative immune checkpoints: LAG-3, TIM-3 and TIGIT

Apart from PD-1/PD-L1/CTLA-4, other inhibitory IC regulate T cell response and might influence IO resistance mechanism. Blocking these additional IC has proven highly efficient in preclinical and clinical studies as monotherapy or in combination with PD-1/PD-L1 inhibitors. The following IC have been investigated:

Lymphocyte activation gene 3 (LAG-3 or CD223) is expressed on various immune cells (Fig. 2). LAG-3 positive T cells bind to ligands such as FGL1 expressed by cancer cells [76], which inhibits activation and cytokine secretion via indirectly blocking of TCR signaling [77]. Studies showed significant co-expression of LAG-3 and PD-1 on TILs [78, 79], with PD-1 marking a range of exhaustion phenotypes in T cells, from mild to anergic, while LAG-3 predominantly marks severely exhausted PD-1 positive CD8^+^ T cells. Hence, LAG-3 synergizes with other IC, particularly PD-1, and dual IC blockage with an anti-LAG3 antibody (e.g. IMP321, relatlimab) *plus* a PD-1/PD-L1 inhibitor has revealed promising preclinical results in different tumor entities and numerous clinical phase I/II trials are currently ongoing [77]. A melanoma study (NCT01968109) presented preliminary efficacy of relatlimab *plus* nivolumab in LAG-3 positive tumors after progression on PD-1/PD-L1 inhibitors. Further phase I/II studies in NSCLC are ongoing as upfront IO combination or in the resistance situation (NCT02750514, NCT02817633).

Similar to LAG-3, the T cell immunoglobulin mucin-3 (TIM-3) negatively regulates T cell activation (Fig. 2). Even though TIM-3 biology is context-dependent, TIM-3 acts as an IC in severely exhausted CD8^+^ T cells. Here, TIM-3 ligands such as galectin-9, HMGB1 or CEACAM-1, expressed by cancer cells, activate TIM-3 and promote T cell anergy [80, 81]. Based on positive preclinical results for anti-TIM-3 antibodies, several clinical trials are ongoing, testing anti-TIM-3 monotherapy or in combination with PD-1/PD-L1 inhibitors [82]: Preliminary results from the phase I Amber trial (NCT02817633) testing anti-TIM3 antibody TSR-022 in combination with a PD-1 inhibitor showed increased clinical activity in anti-PD-1 refractory NSCLC and melanoma. A phase I trial (NCT03099109) investigating anti-TIM3 antibody LY3321367 monotherapy showed preliminary anti-tumor activity and a phase I trial (NCT03708328) investigates a bi-specific antibody targeting TIM-3 and PD-1 in advanced or metastatic solid tumors.

Lastly, T cell immunoglobulin (Ig) and immunoreceptor tyrosine-based inhibitory motif (ITIM) domains (TIGIT) is a lymphocyte-specific transmembrane glycoprotein receptor (Fig. 2). As a co-inhibitory receptor, it exerts direct immunosuppressive effects on these cells through binding to CD155 (and with less affinity CD112) on APC or target cells. TIGIT is weakly expressed in naïve cells but can be rapidly induced in response to inflammatory stimuli [83]. It has been shown to impact many steps of the cancer immunity cycle (reviewed in [83]) and TIGIT inhibition can enhance anti-tumor T cell responses (CITYSCAPE trial), as discussed in later.

### IO combined with Anti-Angiogenic Drugs (AAD)

#### Background and rationale for the combination

VEGF is the key promoter of hypoxia-driven neo-angiogenesis in the TME and also serves as important immunosuppressive molecule. Furthermore, VEGF inhibition has the ability to normalize tumor vasculature and restore chaotic blood flow, thus reducing tumor hypoxia and facilitating immune cell infiltration [84]. These mechanisms depict the functional basis of synergistic AAD and IO effects. Positive preclinical investigations in different cancer entities build a strong rationale for further clinical studies.

#### Clinical translation

Therapeutic combinations of AAD and IO have already been approved for RCC and endometrial cancer. In non-squamous NSCLC, the IMpower150 trial showed an OS benefit for the first-line quadruple (atezolizumab/bevacicumab/carboplatin/paclitaxel) therapy versus AAD/doublet-chemotherapy with a particular benefit in patients with EGFR-mutant/ALK-positive tumors or baseline hepatic metastases [85]. The observed benefit in patients with liver metastasis adds on to previous investigations by Sandler et al. [86] that showed benefit of the AAD/chemotherapy combination, suggesting an organotypic vascular phenotype predisposing to AAD sensitivity. To clinically validate these combinational approaches, deeper investigation of synergistic anti-tumor functions and related toxicity is required. Regarding currently ongoing studies and the basic concepts we refer to other comprehensive reviews [87, 88].

### IO and radiotherapy

#### Background and rationale

Radiation acts cytotoxic by inducing caspase-driven genomic and mitochondrial DNA fragmentation in tumor cells, promoting the release of cytochrome c from mitochondria to activate caspase 9 (CASP9) to ultimately initiate intrinsic apoptosis. Also, radiation alters the inflammatory TME by activating cytosolic DNA sensing pathways (particularly c-GAS-cGAMP-STING cascade, discussed below) in DC [89], possibly also endothelial cells (EC) [90], resulting in IFN I production and activation of anti-cancer immune responses [89]. Irradiated tumor cells often fail to activate DNA sensing pathways to produce IFN I and this barrier most likely depends on CASP9, as blocking radiation-induced CASP9 with a pan-caspase inhibitor emricasan activates tumor-intrinsic type I IFN production, thereby promoting anti-tumor immune responses. However, in this study CASP9 inhibition resulted in PD-L1 upregulation by tumor cells as adaptive resistance strategy. Thus, combinational blockage by emricasan *plus* PD-L1 inhibitor enhanced radiation effects [91].

#### Clinical translation

The additive effect of radiotherapy and IO was investigated in the phase III PACIFIC trial. A long-term survival benefit was seen with PD-L1 inhibitor durvalumab versus placebo when used as consolidation therapy in patients with stage III unresectable NSCLC, who did not have disease progression after concurrent chemoradiotherapy [92].

### DNA damage inhibitors (PARP inhibitors)

#### Background and rationale

DNA damage occurs frequently during cell replication and cells have evolved various DNA Damage Response (DDR) pathways to repair damaged DNA, which when accumulating would lead to cell cycle arrest or apoptosis [93]. One DDR mechanisms involves the poly ADP-ribose polymerase (PARP), a key protein repairing single-strand DNA breakages. Therapeutic PARP inhibition triggers effective anti-cancer immune responses [94]. Double-strand DNA breaks are repaired by homologous recombination (HR). The germline BRCA1/2 genes are involved in HR mechanism and their mutation may lead to HR deficiency (HRD) [95]. HRD alone does not always induce apoptosis as other repair mechanisms can prohibit accumulation of damaged DNA. However, impairing two DDR mechanisms by adding PARPi to HR-deficient cells can lead to cell death (synthetic lethality) [95].

#### Clinical translation

PARP inhibitors (PARPi) are well established in the treatment of BRCA-mutated breast (Olaparib, Talazoparib) and ovarian cancer independent of HRD status (Olaparib, Niraparib, Rucaparib), being highly associated with sensitivity to platinum-based chemotherapy [96].

The BRCA-proficient NSCLC is not clinically responsive to PARPi monotherapy. However, numerous clinical studies showed synergistic effects of PARPi and IO in several solid BRCA-proficient malignancies [97]. As observed preclinically, PARPi induces genetic instability, increases TMB and neoantigen burden via DDR deficiency and may be involved in PD-L1 upregulation by cancer cells [97, 98]. This enhanced tumor immunogenicity explaining potential synergy with IO [97, 99, 100].

Following these encouraging investigations, combinational IO/PARPi NSCLC studies are ongoing: The phase II Hudson umbrella trial (NCT03334617) investigates durvalumab *plus* olaparib in PD-1/PDL-1 refractory patients. The phase II Jasper trial (NCT03308942) studies first-line Niraparib *plus* a PD-1 inhibitor in PD-L1 positive patients progressive on chemotherapy. Results have not been released, however preliminary data from other tumor entities are promising [101, 102]. Lastly, an ongoing phase III trial (NCT02106546) investigates first-line veliparib *plus* chemotherapy versus placebo *plus* chemotherapy in advanced or metastatic NSCLC patients.

Altogether, combining PD-1/PD-L1 inhibitors with PARPi is preclinically active in BRCA-proficient tumors and numerous clinical investigations in NSCLC are ongoing.

### STING agonists

#### Background and rationale

The *cGAS-STING pathway* has been identified as key intracellular pathway bridging anti-cancer innate and adaptive immunity [103]. Stimulator of Interferon Genes (STING) is a cytosolic protein of phagocytic immune, endothelial and cancer cells (Fig. 2) that gets activated by the enzyme cyclic-GMP-AMP synthase (cGAS) via the cyclic dinucleotide (CDN) second messenger cGAMP. The STING pathway senses cytosolic DNA (self or foreign e.g. cancer-derived DNA) and, via activation of numerous downstream signals, induces IFN I IFN-ß. IFN-ß plays a major role in priming adaptive immunity, including activation and recruitment of CD8^+^T cells and promoting DC migration and maturation, thus enhancing anti-tumor immune responses [103, 104]. Cancer cells can downregulate STING activity to evade immune-mediated apoptosis [105].

#### Clinical translation

Based on this understanding, STING agonists, including STING-binding molecules and CDN derivatives, are being developed as novel cancer therapeutics. Preclinical studies showed dramatic anti-cancer effects of intratumorally (i.t.) applied STING agonist [90, 106–108]. Importantly, the STING induced increase in CD8^+^ T cells at the tumor site can enhance concomitant anti-PD-1 therapy effect [109, 110]. The synthetic STING agonist *ADU-S100* is currently under investigation in clinical phase I/II trials (NCT02675439, NCT03937141) as i.t. monotherapy or in combination with ICI in advanced solid tumors or lymphoma. A first-in-human study (NCT03010176) of STING agonist MK1454 as i.t. monotherapy or together with pembrolizumab in advanced solid tumors or lymphomas showed encouraging results with PR in 24% of patients and substantial tumor size reduction (83% of both injected and non-injected target lesions).

In conclusion, i.t. STING agonists may evolve as potent combination to ICI treatment by “boosting” cancer-directed immune responses and sensitizing tumor cells to ICI.

### IDO inhibitors

#### Background and rationale

Tryptophan catabolism, involving the key enzymes indoleamine 2,3-dioxygenase 1 and 2 (IDO1 and 2) and tryptophan-2,3-dioxygenase (TDO2) is a critical metabolic pathway in cancer progression. IDO is IFN-induced in cancer, stromal non-immune and immune cells that metabolizes tryptophan to kynurenine. Its overexpression has immunosuppressive functions by depleting tryptophan and increasing kynurenine in the TME. Indeed, kynurenine accumulation and tryptophan depletion promotes the generation of Tregs and MDSCs, and inhibits T_eff_ proliferation and activation [111]. IDO1 upregulation has been demonstrated in numerous cancer types, including NSCLC, and associates with poor prognosis and IO resistance [56]. Various preclinical studies demonstrated increased T cell proliferation and tumor infiltration as well as IL-2 upregulation upon IDO1 inhibition (reviewed in [112]). Although investigated to a lesser extent, TDO2 exerts similar immunosuppressive functions and enhanced expression has been shown in NSCLC [56].

#### Clinical translation

IDO1 inhibitors (IDO1i) have been tested in multiple phase I/II trials in combination with PD-1/PD-L1/CTLA-4 inhibitors with promising results (reviewed in [113]). However, the first large phase III ECHO-301 trial evaluating the selective IDO1i epacadostat in combination with pembrolizumab in advanced melanoma was terminated early as the primary endpoint (improved PFS compared to pembrolizumab) was not reached [114]. Many flaws, such as insufficient dosing, lack of pharmacodynamic surrogates for drug efficacy and testing in an unselected patient population (without prior IDO testing) limit the value of the trial. Moreover, the inclusion of patients pre-treated with CTLA4- or BRAF inhibitors might explain the beneficial lack of selective IDO1i, as these therapies enhance TME levels of IDO1 and the compensatory molecules TDO2 and IDO2, which may have increased cytotoxic TIL and IFN-γ, hence impeding the effect of concomitant PD-1 blockade [56]. Still, the scientific rationale of IDO1i is solidly grounded and further clinical investigation is ongoing. Other drug combinations might evolve as efficient partners for IDO1i, e.g. CTLA-4 inhibitors, STING agonists or radio-chemotherapy [115].

### Arginase inhibitors

#### Background and rationale

Arginine is a semi-essential amino acid critical for lymphocyte proliferation and function. The enzymes arginase 1 and 2 (ARG1/2) regulate extracellular arginine availability by converting arginine to ornithine and urea. High ARG1/2 expression and activity has been shown in various cancer types including NSCLC [116] and associates with poor prognosis. Within the TME, ARG is mainly produced by myeloid cells (i.e. MDSC, macrophages) in response to local stimuli (e.g. immunosuppressive cytokines, hypoxia, acidosis). ARG impedes T cell function e.g. by downregulation of TCR CD3ζ chain, lowers Th1 cytokine production (IFN-γ, TNF-β) and inhibits T cell proliferation and differentiation [117]. Thus, therapeutic ARG inhibition may enhance anti-tumor immunity. Contrarily, preclinical studies implicated that arginine deprivation by using recombinant human ARG can induce apoptosis in some tumors, including NSCLC.

#### Clinical translation

ARG inhibitors have entered clinical trials and most substances competitively target ARG1 and ARG2. In advanced or metastatic solid cancers including NSCLC a phase I/II study (NCT02903914) investigates the small molecule INCB001158 alone or in combination with pembrolizumab. First results from CRC show manageable AEs and clinical responses. The substance OATD-02 is a selective ARG1/2 inhibitor and has shown significant anti-tumor immunity in preclinical tumor models alone or in combination with PD-1 or IDO1i.

### Epigenetic modulators + IO

#### Background and rationale

Epigenetic-modulating drugs like 5-azacitidin (DNA hypomethylating agent) and entinostat (class I HDAC inhibitor) are well established in hematology. In addition to reactivating expression of epigenetically silenced tumor suppressor genes in cancer cells, these drugs may also selectively inhibit MDSC by induction of viral mimicry via inducing retrotransposon-derived dsRNA. This increases tumor foreignness through enhanced neoepitope expression, as well as it upregulates genes related to immune-evasion, such as B2M. In preclinical models, the combination of epigenetic modulators and PD-1 inhibitors has shown major therapeutic effects [54, 118].

#### Clinical translation

Based on these investigations, numerous phase I/II clinical trials in various solid tumor entities have been initiated, including NSCLC. Though interim analysis (e.g. ENCORE 601 trial) showed promising results, most of these studies are currently still ongoing [119].

### Adenosin-signaling pathway (CD73)

#### Background and rationale

Adenosine is an effective endogenous immunosuppressive mediator in normal and cancerous tissues. It gets either excreted by stressed or injured cells or generated via a multi-staged pathway from extracellular adenosine-triphosphate (ATP) through dephosphorylation of adenosine-monophosphate (AMP) by the enzyme CD73 [120]. In the TME both CD73 and adenosine are widely expressed on a variety of cells (Fig. 2). Adenosine acts via binding the A2a receptor (A2aR) (expressed on lymphocytes, myeloid and NK cells, CAF, EC), provoking i.e. Treg and MDSC accumulation, T_eff_ and NK cell inhibition or CAF proliferation, thereby fostering a tumorigenic TME. CD73 expression and consequently adenosine generation is regulated via complex molecular pathways, including HIF-1alpha, MAPK, mTOR, TGF-beta [120]. Some tumors overexpress CD73 as a possible immune-evading strategy while others do not. CD73 upregulation has been associated with an inferior outcome in NSCLC [121], and in preclinical cancer models, high CD73 expression correlated with a better response to CD73 blockade [122]. In NSCLC, high A2aR expression correlated with lower CD4^+^ and CD8^+^ T cell activation and lower PD-L1 expression [123].

#### Clinical translation

Therapeutic attempts have focused on inhibiting adenosine production by targeting CD73 or interfering with adenosine signaling by targeting A2aR. Different anti-CD73 antibodies have entered clinical trials as monotherapy or in combination with ICI: The anti-CD73 antibody oleclumab *plus* durvalumab is being tested in phase II studies in locally advanced or metastatic ICI-refractory (COAST, NCT03822351; HUDSON, NCT03334617, respectively) or as neo-adjuvant therapy in resectable (NeoCOAST, NCT03794544) NSCLC. Concerning A2aR antagonists the two oral small molecules cifroadenant (CPI-444) and AZD4635 are currently under investigation in phase I studies (NCT03337698 and NCT02740985, respectively) alone or in combination with PD-L1 inhibitors. NSCLC-regarding results of both studies have not been released yet.

### Chemokine receptor antagonists: CCR4 and CXCR2 inhibitors

#### Background and rationale

The CC chemokine receptor type 4 (CCR4) is expressed on Treg and other circulating/tumor-infiltrating T cells and binding of TME-derived ligands (CCL17, CCL22) to CCR4 promotes recruitment of immunosuppressive Treg. Therapeutic Treg depletion may alleviate the suppression of anti-tumor immunity and hence synergize with PD-1 inhibition, as also suggested by a preclinical study [55]. Furthermore, the CXCL5/CXCR2-axis mediates myeloid cell recruitment and CXCR2 blockade significantly reduced presence of MDSC in murine tumors [124]. CCR4 and CXCL5 expression has been associated with poor prognosis in various cancer types including NSCLC [125, 126].

#### Clinical translation

The monoclonal anti-CCR4 antibody mogamulizumab exerts Treg-depleting effects and is FDA-approved for refractory T cell lymphoma. First results from phase I solid tumor trials in combination with PD-1/PD-L1/CTLA-4 inhibitors suggest an acceptable safety profile [127, 128] and antitumor effects of mogamulizumab/nivolumab in a small NSCLC subgroup [127]. Different CXCR2 antagonists are getting investigated preclinically and clinically (reviewed in [124]), acting as neutrophil-directed immunotherapy. A phase II trial is currently testing the selective CXCR2 antagonist navarixin (MK-7123) together with pembrolizumab in advanced solid tumors including NSCLC (NCT03473925). Although only at the beginning of an understanding, these data pinpoint to possible future chemokine-targeted therapies in cancer.

### CSF1R antagonists

#### Background and rationale

Polarization of TAM to the pro-tumorigenic M2 phenotype is promoted by binding of tumor cell-derived M-CSF to CSF1R on TAM. Anti-CSF1R antibodies can deplete TAM, however clinical studies failed to show potent anti-tumor effects of the monotherapy (e.g. NCT01494688). A study by Kumar et al. showed that CSF downregulates granulocytic chemokine (e.g. CXCL1/2) production by CAF and that anti-CSF1 antibodies hence promote TME infiltration by immunosuppressive MDSC. Inhibition of both CSF1R and CXCR2 decreased TME infiltration by TAM and MDSC, significantly reduced tumor growth and enhanced the effect of PD-1 inhibitor [129].

#### Clinical translation

Numerous ongoing preclinical studies are testing CSF1R antagonists with different IO partners. In advanced NSCLC, two phase I trials (NCT03502330, NCT02526017) are currently investigating the CSF1R antagonist cabiralizumab in combination with an anti-CD40 mAb or nivolumab, respectively. Unfortunately, a recent phase II trial (NCT03336216) testing cabiralizumab *plus* nivolumab in advanced pancreatic cancer failed its primary endpoint.

### RIG-I

#### Background and rationale

Retinoic acid Inducible Gene 1 (RIG-I) is a cytosolic RNA receptor ubiquitously expressed in most human body cells and is known for its major role in antiviral immune defense by inducing pyroptosis. RIG-I is also expressed in cancer cells, acting pro-inflammatory by expressing INF I and other cytokines [130]. In preclinical models, systemically applied RIG-I agonists were able to inhibit tumor growth via induction of immunogenic cancer cell death [131–133].

#### Clinical translation

Intratumoral application of the selective RIG-I agonist RGT100 was investigated in a small phase I/II first-in-human study (NCT03065023) in advanced or recurrent cancer (*n* = 15). There were no dose-limiting toxicities, especially as only minimal systemic exposure was found after i.t. application. Interestingly, systemic chemokine elevation and INF-associated gene expression were detected. RIG-I agonists are only at the starting point of clinical applicability. Therapeutic challenges include the development of highly selective agonists due to ubiquitous RIG-I expression and to avoid uncontrolled cytokine release.

### Fibroblast Activation Protein (FAPα)

#### Background and rationale

The immunosuppressive activity of CAF can be hampered by blocking cell surface markers and most experience has been gathered with fibroblast-activation protein α (FAPα), a common but non-selective CAF marker in many cancer types [134]. In a mouse model, FAPα-blockade resulted in tumor growth inhibition and stromal reduction of myofibroblasts and vasculature in lung and colon tumors [135]. Other preclinical strategies include FAPα-targeted oncolytic adenovirus-vaccination [136] or FAPα-targeted chimeric antigen receptor T cell (CAR-T) [137].

#### Clinical translation

A recent pioneer study investigated the use of a bispecific antibody (RO6874281) consisting of an interleukin-2 variant (IL-2v) domain that binds the IL-2 receptor on immune cells and a FAPα-specific domain, which tracks the antibody-drug conjugate inside the tumor and reduces efflux. RO6874281 showed an acceptable safety profile and displayed monotherapy activity in tumor types not previously reported to respond to IL-2 [138] A phase II trial (NCT02627274) of RO6874281 together with atezolizumab is currently ongoing. CAFs and their immunosuppressive network present an interesting therapeutic target, however non-specificity of molecular markers incorporates a major hurdle and needs further exploration.

## Discussion

In this article, we discussed relevant immunomodulatory pathways imprinted within the TME that fundamentally impact the evolution of IO resistance in NSCLC and summarized novel therapy approaches targeting many of these alterations. Considering that the majority of NSCLC patients eventually progress on IO therapy, combinational or multimodal treatment approaches are an unmet medical need.

The mechanisms underlying IO efficacy are still incompletely understood. Factors such as the dynamic cellular composition and heterogeneity of immunogenic and metabolic pathways within the TME, as well as the mutational load driving tumor immunogenicity, all contribute to IO effectiveness and evolution of resistance mechanisms.

The hallmarks of carcinogenesis are significantly influenced not only by cancer cell-intrinsic mechanisms but also by the different stromal cell populations [139]. The heterogeneity and complexity of the stromal TME and associated pathway activities and resistance patterns were particularly highlighted in lung cancer by recent high-resolution profiling [2]. However, it is likely that many of the here described TME alterations are universally apparent across different tumor entities and most preclinical studies and early-phase IO trials include several, mostly solid cancer types. At the current state of knowledge, no NSCLC-specific molecular target has been identified yet. Nevertheless, differences in the relative abundances of tumor infiltrating immune and stromal cells as well as the mutational burden do exist across different tumor entities [140].

Many of the discussed novel treatment approaches either aim to inhibit intrinsic immunosuppressive (IDO, CD73/adenosine, VEGF, CCR4, CXCR2, arginase) or promote proinflammatory/immunogenic (STING, RIG-I, PARP) pathways. Combinations of these targeted approaches with different ICI are often synergistic and may evolve as promising strategies to overcome IO resistance. Moreover, dual ICI therapy with PD-1/CTLA-4 antibodies may boost intrinsic anticancer immunity and has previously been translated into clinical OS benefit (see CheckMate227). Combinations of PD-L1 and alternative IC (e.g. LAG-3, TIM-3, TIGIT) have shown promising results in phase I trials.

Concerning biomarkers, PD-L1 is still considered the most robust biomarker in NSCLC, even though in many cases its predictive power is insufficient. Thus, the need for further, more complex biomarker-signatures that help to optimize patient selection for the different IO strategies is immense. A priori identification of resistance mechanisms in order to initiate targeted therapies upfront will depict a major challenge. In-depth tumor analysis including whole-genome sequencing, single cell RNA-sequencing, multidimensional flow cytometry or epigenetics might be implemented in the future as to find individualized treatment strategies.

## Conclusion

IO therapy induces a wide range of cellular and molecular alterations in the TME and resistance mechanisms are only partially understood. However, as research is rapidly growing, numerous targets have been identified that may inhibit or override IO resistance. With positive results from many clinical trials, these novel IO combinational approaches pose a promising outlook for future therapies that improve clinical outcome and patient survival.

## Data Availability

The datasets generated and/or analysed during the current study are available in the Scope repository, http://scope.aertslab.org/#/Bernard_Thienpont/*/welcome.

## Abbreviations

AAD: Anti-angiogenic drugs
AE: Adverse events
AMP: Adenosine-monophosphate
ARG: Arginase
ATP: Adenosine-triphosphate
B2M: β2 microglobulin
CAF: Cancer-associated fibroblasts
CCR4: CC chemokine receptor type 4
CDN: Cyclic dinucleotide
cGAS: Cyclic-GMP-AMP synthase
CRC: Colorectal cancer
CSF: Colony stimulating factor
CTLA-4: Cytotoxic T lymphocyte antigen 4
DDR: DNA Damage Response
FAPα: Fibroblast-activation protein
αHEV: High endothelial venules
HR: Homologous recombination
HRD: HR deficiency
IC: Immune checkpoints
ICI: Immune checkpoint inhibitors
IDO1: Indoleamine 2,3-dioxygenase
IFN: Interferon
IHC: Immunohistochemistry
IL-2v: Interleukin-2 variant
IO: Immunotherapy
LAG-3: Lymphocyte activation gene 3
LPS: Lipopolysaccharide
M-CSF: Macrophage-colony stimulating factor
MDSC: Myeloid-derived suppressor cells
MHC: Major histocompatibility complex
MMR: Mismatch repair
NSCLC: Non-small cell lung cancer
ORR: Objective response rate
OS: Overall survival
PARP: Poly ADP-ribose polymerase
PARPi: PARP inhibitors
PD-1: Programmed death receptor 1
PD-L1: PD-ligand 1
RCC: Renal cell carcinoma
RIG1: Retinoic acid Inducible Gene 1
STING: Stimulator of Interferon Genes
TAM: Tumor associated macrophage
TCR: T cell receptor
TDO2: Tryptophan-2,3-dioxygenase
TEC: Tumor endothelial cells
TIGIT: T cell immunoglobulin and immunoreceptor tyrosine-based inhibitory motif domains
TIL: Tumor infiltrating T lymphocytes
TIM-3: T cell immunoglobin mucin-3
TLR: Toll-like receptor
TLS: Tertiary lymphoid structures
TMB: Tumor mutational burden
TME: Tumor microenvironment
VEGF: Vascular endothelial growth
